# Brain MRI findings in paediatric genetic disorders associated with white matter abnormalities

**DOI:** 10.1111/dmcn.16036

**Published:** 2024-07-30

**Authors:** Jaakko H. Oikarainen, Oula A. Knuutinen, Salla M. Kangas, Elisa J. Rahikkala, Tytti M.‐L. Pokka, Jukka S. Moilanen, Reetta M. Hinttala, Päivi M. Vieira, Johanna M. Uusimaa, Maria H. Suo‐Palosaari

**Affiliations:** ^1^ Medical Research Center Oulu University of Oulu and Oulu University Hospital Oulu Finland; ^2^ Research Unit of Health Sciences and Technology University of Oulu Oulu Finland; ^3^ Department of Neurosurgery Oulu University Hospital Oulu Finland; ^4^ Biocenter Oulu University of Oulu Oulu Finland; ^5^ Department of Clinical Genetics Oulu University Hospital Oulu Finland; ^6^ Research Unit of Clinical Medicine University of Oulu Oulu Finland; ^7^ Clinic for Children and Adolescents Oulu University Hospital Oulu Finland; ^8^ Department of Diagnostic Radiology Oulu University Hospital Oulu Finland

## Abstract

**Aim:**

To describe the specific brain magnetic resonance imaging (MRI) patterns of the paediatric genetic disorders associated with white matter abnormalities in Northern Finland.

**Method:**

In this retrospective population‐based longitudinal study, brain MRI scans accumulated from 1990 to 2019 at Oulu University Hospital, Finland, were assessed. Inclusion criteria were defined as leukodystrophies or genetic diseases with significant white matter abnormalities that did not meet the criteria for leukodystrophy, at least one brain MRI, and age under 18 years at diagnosis.

**Results:**

A total of 83 patients (48 males, 35 females) were found with 52 different diseases. The median age at the time of the brain MRI was 22 months (interquartile range [IQR] = 46 months). In 72 (87%) of the children, brain MRIs revealed abnormal findings, including cerebral white matter abnormalities (*n* = 49, 59%), brainstem signal abnormalities (*n* = 28, 34%), thinning of the corpus callosum (*n* = 30, 36%), delayed myelination (*n* = 11, 13%), and permanent hypomyelination (*n* = 9, 11%).

**Interpretation:**

Symmetrical and bilateral white matter signal patterns of the brain MRI should raise suspicion of genetic disorders when the clinical symptoms are compatible. This study illustrates brain imaging patterns of childhood‐onset genetic disorders in a population in Northern Finland and improves the diagnostic accuracy of rare genetic disorders.

## Graphical abstract

Plain language summary: https://onlinelibrary.wiley.com/doi/10.1111/dmcn.16100

This original article is commented by Wolf et al. on pages 143–144 of this issue.

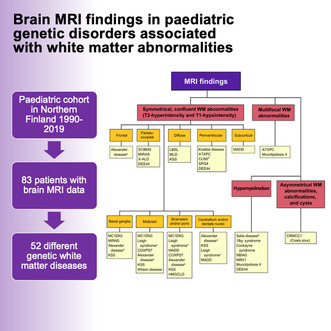

**Abbreviations**
CRMCC1: cerebroretinal microangiopathy with calcifications and cysts
FINCA: fibrosis, infection susceptibility/immunodeficiency/intellectual disability, neurodevelopmental disorder/neurodegeneration, and chronic anaemia/cerebral angiomatosis
gLE: genetic leukoencephalopathy
GWMD: genetic white matter disorder
SCBMS: seizures, cortical blindness, and microcephaly syndrome

**What this paper adds**

- Symmetrical and bilateral brain white matter signal patterns suggest genetic disorders.
- Delayed myelination and permanent hypomyelination are uncommon in genetic brain disorders.

Genetic white matter disorders (GWMDs) consist of a large and heterogeneous group of diseases affecting the white matter of the central nervous system.^1^ GWMDs are often diagnosed during childhood, but they may occur later in adulthood.^2^ Numerous genetic and metabolic errors have been identified in the pathogenesis of GWMDs.^1^, ^3^ They can be further categorized into classic leukodystrophies and genetic leukoencephalopathies (gLEs), depending on whether the central nervous system white matter involvement is considered to be primary or secondary.^4^ In 2017, van der Knaap and Bugiani described a new approach to the classification of leukodystrophies based on cellular pathology that takes into account the primary involvement of any white matter component.^5^ Brain magnetic resonance imaging (MRI) is a crucial application in the diagnostics of GWMDs, as it is highly sensitive in detecting white matter abnormalities.^1^ Many GWMDs, especially cases of classic leukodystrophies, have typical or pathognomonic imaging findings in MRI, which have led to use of the pattern recognition approach as a primary diagnostic method.^6^, ^7^, ^8^ Brain MRI interpretation and pattern recognition may help to target specific biochemical or single‐gene tests.^1^, ^3^, ^7^, ^8^, ^9^

The prevalence of GWMDs in Northern Finland differs from other parts of Europe and the rest of the world, most likely because of the Finnish disease heritage.^10^ The population in this retrospective longitudinal study represents unique prevalence and brain MRI findings among GWMDs.^11^ The aim of this study was to evaluate specific radiological patterns in defined or recently characterized GWMDs in this distinct population. Furthermore, radiological findings in recently identified GWMDs were compared with previous literature.

## METHOD

GWMDs were defined as leukodystrophies or genetic leukoencephalopathies according to the International Statistical Classification of Diseases and Related Health Problems, 9th and 10th Revisions, and the previously described definition by Vanderver et al.,^4^ where genetic leukoencephalopathies accounted for disorders with significant, if not primary, white matter abnormalities that did not meet the criteria for inclusion as a leukodystrophy. According to this classification, gLEs were divided into mitochondrial and other gLEs based on genetic and biochemical data. Furthermore, patients with a recently described genetic diagnosis and noted white matter signal abnormalities on brain MRI were added to the cohort. These syndromes are seizures, cortical blindness, and microcephaly syndrome (SCBMS); fibrosis, infection susceptibility/immunodeficiency/intellectual disability, neurodevelopmental disorder/neurodegeneration, and chronic anaemia/cerebral angiomatosis (FINCA disease); cerebroretinal microangiopathy with calcifications and cysts 1 (CRMCC1); and ataxia‐pancytopenia syndrome (ATXPC).^12^, ^13^, ^14^, ^15^, ^16^

This study was performed at Oulu University Hospital, Finland, as part of the Genetics of Northern Finland Leukoencephalopathies and Leukodystrophies study. Oulu University Hospital serves as a tertiary care centre for child neurology in Northern Finland, which had a total population of 736 883 in 2019.^17^ The study population included all children diagnosed and treated with GWMDs at the Department for Children and Adolescents of Oulu University Hospital between 1990 and 2019. Epidemiological, clinical, and genetic data from this population were collected and published previously, consisting of 80 patients with 49 different GWMDs.^11^ Furthermore, three additional patients who fulfilled all the inclusion criteria were recruited. In the final analysis, 83 patients and 52 different GWMDs were included in the study. Cases with known GWMDs were identified from the patient registry by their diagnoses, whereas patients with recently described GWMDs were identified by the physicians involved in this study.

The inclusion criteria for this study were patients under 18 years of age at the time of diagnosis or at the time of clinical evaluation, a diagnosis of GWMD or genetic disorders associated with white matter abnormalities, and at least one MRI scan available. Exclusion criteria were patients older than 18 years, patients living outside the tertiary catchment area of Oulu University Hospital, and patients with no MRI data available. Clinical data were collected from patient records. Radiological data were collected from both the film archives of the hospitals and the digital picture archiving and communication system.

During the period 1990 to 2019, brain MRIs were performed with variable imaging protocols and scanners. The magnet field strengths used were 1 Tesla in 11 (13%), 1.5 Tesla in 25 (30%), and 1.5 Tesla or 3 Tesla in 47 (57%) of the patients. The applied protocols varied depending on clinical aspects. Appropriate and available T1‐ and T2‐weighted and T2 fluid‐attenuated inversion recovery sequences were considered minimum to fulfil the requirements for inclusion in the study.

MRI scans were evaluated by two radiologists (MS‐P, 18 years of experience in radiology and 12 years in paediatric neuroradiology, and JO, 4 years of experience in radiology). Radiological data were collected using a specific form (Appendix S1). For every patient, the state of myelination was marked as normal, delayed, or permanent hypomyelination. Permanent hypomyelination was defined as an unchanged pattern of deficient myelination in two MRIs at least 6 months apart in a child older than 1 year, according to an article published by Schiffmann and van der Knaap.^6^ In a few cases, permanent hypomyelination was established without follow‐up scans if the patient was older than 2 years and presented severely deficient myelination.^6^ Both T1‐ and T2‐weighted images were used to determine the state of myelination in every patient. Signal abnormalities, agenesia or hypogenesia of the structures, swelling, and restricted diffusion were reported separately for the basal ganglia, corpus callosum, midbrain, cerebellum, dentate nuclei, pons, and medulla oblongata. Possible brain atrophy was evaluated by a systematic approach to analyse sulcal and ventricular dilatation and volume loss of gyri by visual rating and comparing serial MRI if available.

### Ethics

The Ethics Committee of the Northern Ostrobothnia Hospital District (EETTMK 68/2018) approved the study, and it was carried out following the Declaration of Helsinki. Patients or their carers gave written informed consent to the research and to publication of the results.

## RESULTS

### Distribution and imaging of GWMDs

Eighty‐three patients fulfilled the inclusion criteria. The cohort presented with subtle male dominance (*n* = 48, 58%). The number of leukodystrophies was 14 (17%), and the number of gLEs was 69 (83%). Of all the gLEs, 17 (20%) diseases were defined as mitochondrial gLEs. Brain MRI was conducted at a median age of 22 months (interquartile range [IQR] = 46 months). In a total of 72 (87%) patients, MRI was considered abnormal. The most common diagnosis in the cohort was X‐linked adrenoleukodystrophy (X‐ALD, *n* = 6, 7%). The second most common disease was a recently described disease, SCBMS (*n* = 4, 5%), which was published in the same year as the previous article concerning genetic aetiologies and clinical features in this cohort.^11^ The most common mitochondrial disorder was mitochondrial encephalopathy, lactic acidosis, and stroke‐like episodes (MELAS, *n* = 3, 4%). Radiological findings for each GWMD in this cohort are presented in detail in Appendix S2.

### Patterns of white matter abnormalities

**FIGURE 1:**
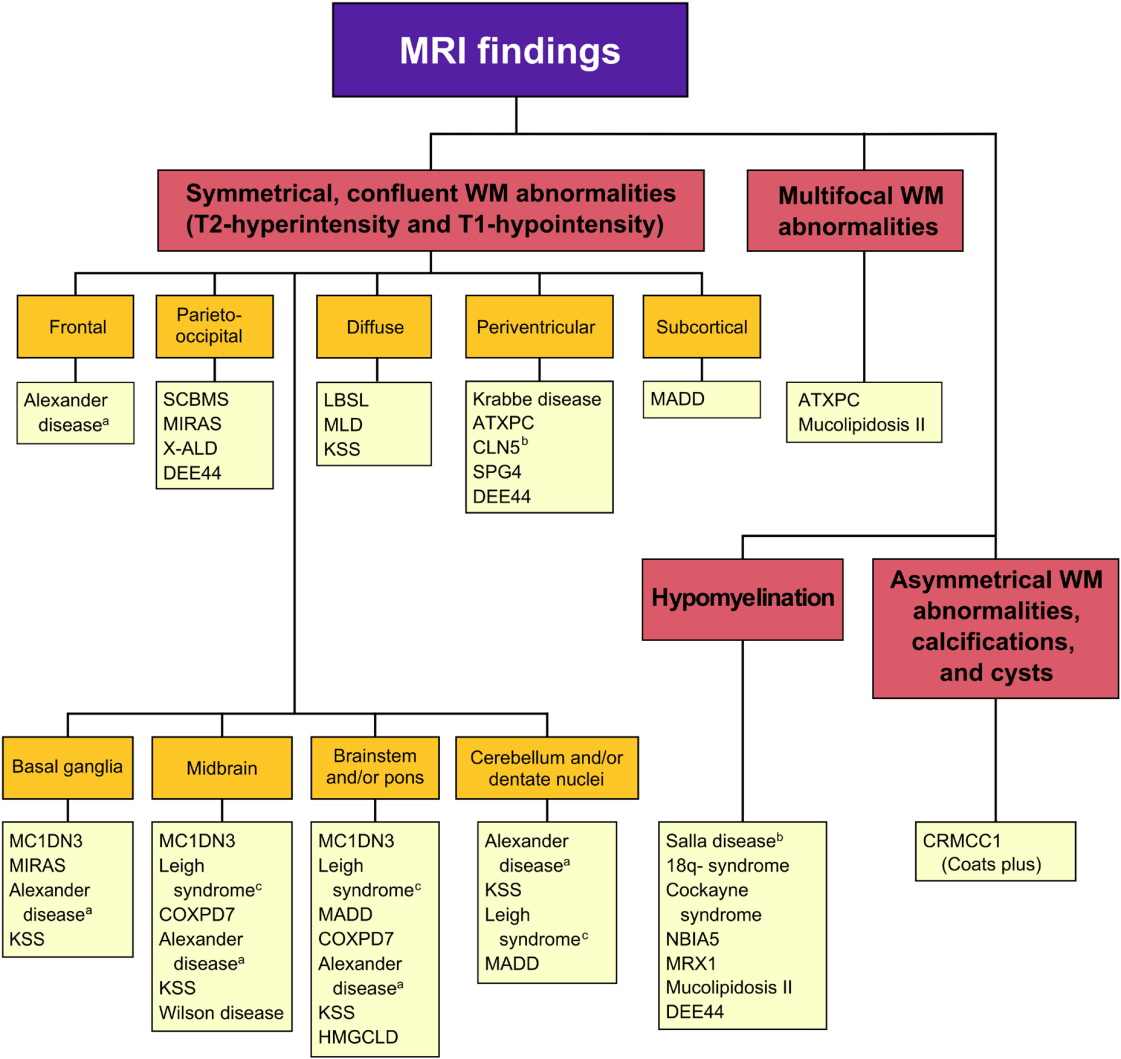
Brain magnetic resonance imaging (MRI) patterns of genetic disorders associated with white matter abnormalities. ^a^ Neonatal subtype. ^b^ Finnish disease heritage. ^c^ SURF1 deficiency. Abbreviations: ATXPC, ataxia‐pancytopenia syndrome; CLN5, neuronal ceroid lipofuscinosis 5; COXPD7, combined oxidative phosphorylation deficiency 7; CRMCC1, cerebroretinal microangiopathy with calcifications and cysts 1 (also known as Coats plus syndrome); DEE44, early infantile epileptic encephalopathy 44; HMGCLD, 3‐hydroxy‐3‐methylglutaryl‐CoA lyase deficiency; KSS, Pearson‐Kearns‐Sayre spectrum (Pearson syndrome or Kearns–Sayre syndrome); LBSL, leukoencephalopathy with brain stem and spinal cord involvement and elevated lactate; MADD, multiple acyl‐CoA dehydrogenase deficiency; MC1DN3, mitochondrial complex I deficiency, nuclear type 3; MIRAS, mitochondrial recessive ataxia syndrome; MLD, metachromatic leukodystrophy; MRX1, X‐linked intellectual disability‐1; NBIA5, neurodegeneration with brain iron accumulation‐5; SCBMS, seizures, cortical blindness, and microcephaly syndrome; SPG4, autosomal dominant spastic paraplegia‐4; X‐ALD, X‐linked adrenoleukodystrophy.

**FIGURE 2:**
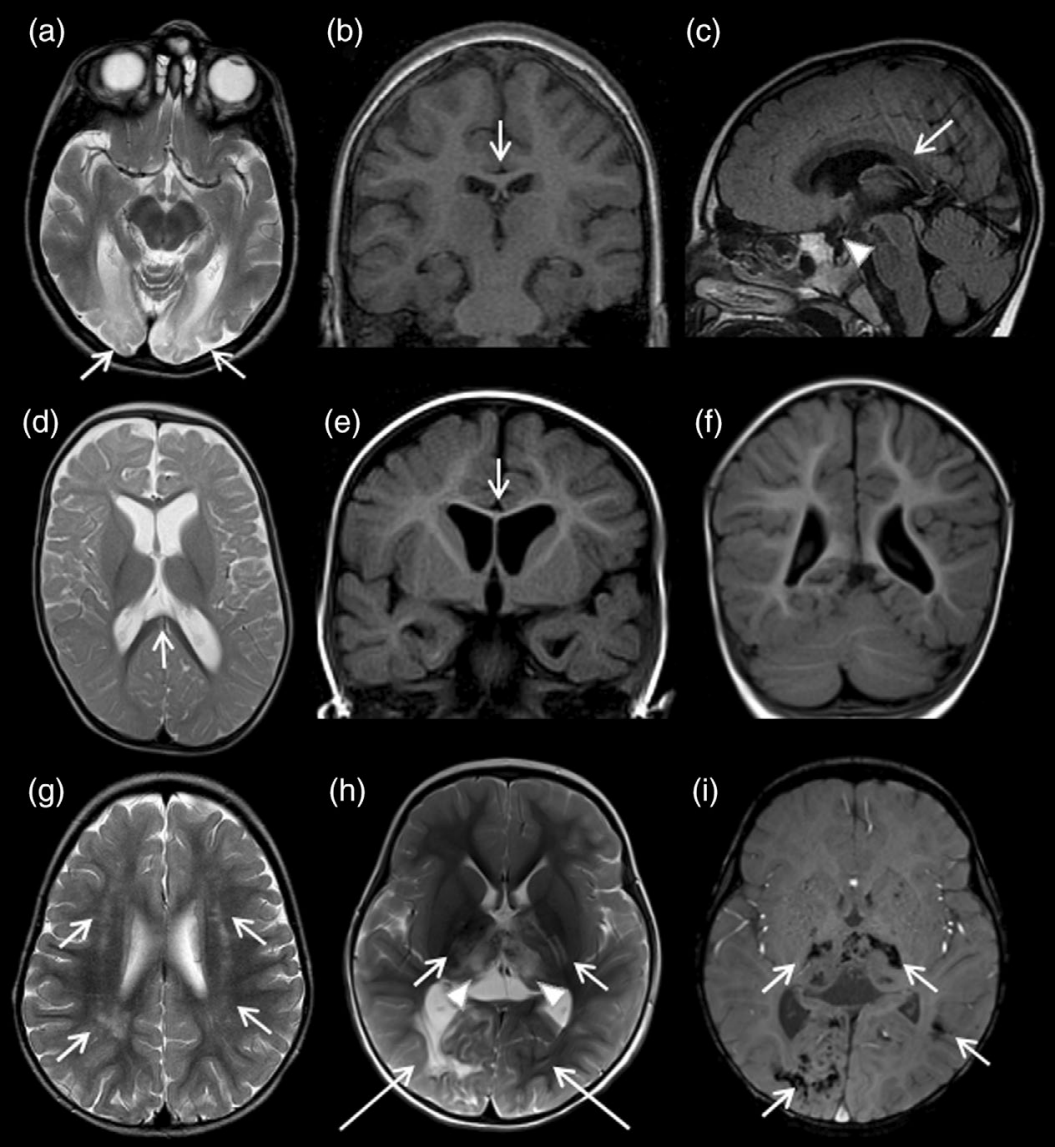
Brain magnetic resonance imaging (MRI) patterns of the uniquely prevalent genetic diseases of this Northern Finnish cohort. Two different patients with seizures, cortical blindness, and microcephaly syndrome at the age of 8 years (a and b) and 4 years (c). Axial T2‐weighted image shows bilateral and symmetrical signal abnormalities, and atrophy in occipital lobes (arrows in a). The corpus callosum shows mild thinning in both patients (arrows in b and c). The optic chiasm is markedly thin (arrowhead in c). Axial T2‐weighted image (d) and coronal T1‐weighted images (e and f) of a 10‐month‐old patient with fibrosis, infection susceptibility/immunodeficiency/intellectual disability, neurodevelopmental disorder/neurodegeneration and chronic anemia/cerebral angiomatosis disease shows thinning of the corpus callosum (arrows in d and e) and marked cerebral cortical atrophy (d–f). A patient with ataxia‐pancytopenia syndrome at the age of 1 year 3 months presents multifocal white matter signal abnormalities in the frontal and parietal lobes on axial T2‐weighted image (arrows in g). A patient with cerebroretinal microangiopathy with calcifications and cysts at the age of 2 years 2 months demonstrates bilateral signal abnormalities in the thalami (arrowheads in h), internal capsule (short arrows in h), and occipital lobes (long arrows in h) on axial T2‐weighted image and marked calcifications in the affected areas on susceptibility weighted imaging (arrows in i).

Brain MRI white matter patterns and other specific findings for the GWMDs of the study cohort are presented in Figures 1 and 2. Cerebral white matter signal abnormalities were detected in 49 (59%) of the patients. The most common distributions of the white matter hyperintense signal abnormality patterns on the T2‐weighted and T2 fluid‐attenuated inversion recovery images were periventricular (*n* = 26, 31%), confluent (*n* = 15, 18%), and subcortical (*n* = 13, 16%). The most common location of the white matter abnormalities was the frontoparietal lobe. Parietal predominance was found in 31 (37%), and frontal predominance was found in 28 (34%) of the patients. Occipital white matter abnormalities were relatively common (*n* = 24, 29%), whereas temporal white matter abnormalities were found in only eight (9%) of the patients.

### Abnormalities of the corpus callosum

**FIGURE 3:**
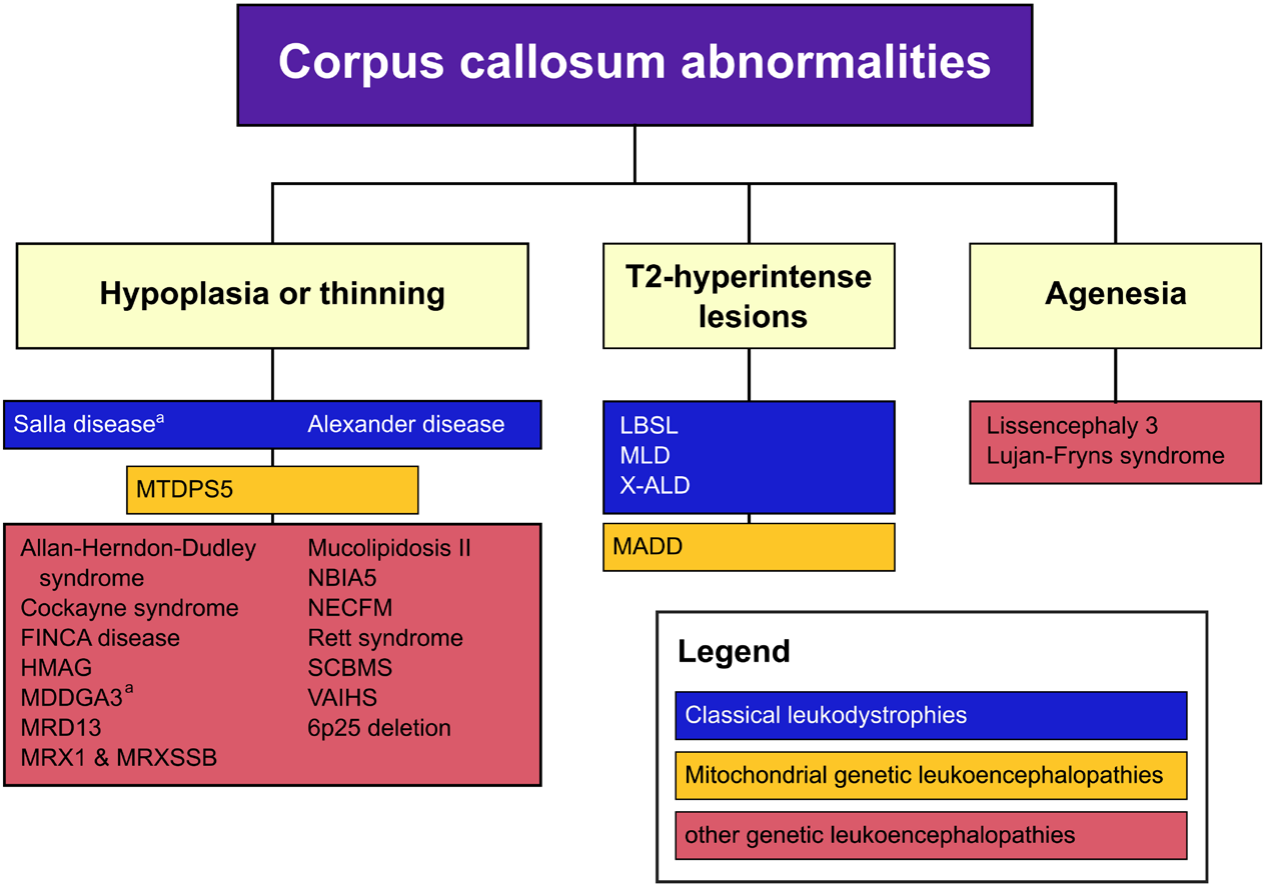
Corpus callosum abnormalities in genetic disorders with white matter abnormalities. ^a^ Finnish disease heritage. Abbreviations: FINCA disease; fibrosis, infection susceptibility/immunodeficiency/intellectual disability, neurodevelopmental disorder/neurodegeneration and chronic anemia/cerebral angiomatosis; HMAG, homocystinuria‐megaloblastic anemia, cblG complementation type; LBSL, leukoencephalopathy with brain stem and spinal cord involvement and elevated lactate; MADD, multiple acyl‐CoA dehydrogenase deficiency; MC1DN3, mitochondrial complex I deficiency, nuclear type 3; MDDGA3, muscle‐eye‐brain disease; MIRAS, mitochondrial recessive ataxia syndrome; MLD, metachromatic leukodystrophy; MRD13, autosomal dominant intellectual disability 13; MRX1, X‐linked intellectual disability‐1; MRXSSB, X‐linked intellectual disability 102; MTDPS5, mitochondrial DNA depletion syndrome; NBIA5, neurodegeneration with brain iron accumulation 5; NECFM, neurodevelopmental disorder with epilepsy, cataracts, feeding difficulties, and delayed brain myelination; SCBMS, seizures, cortical blindness, and microcephaly syndrome; VAIHS, vasculitis, autoinflammation, immunodeficiency, and hematologic defects syndrome; WM, white matter; X‐ALD, X‐linked adrenoleukodystrophy.

All diseases related to the pathology of corpus callosum are presented in Figure 3. Abnormalities of the corpus callosum were common in the study cohort, although most of the abnormalities were subtle, such as thinning or hypoplasia of the corpus callosum. These findings were found in 25 different diseases and in all subtypes of GWMDs in this cohort. Thinning or hypoplasia of the corpus callosum was interpreted in 29 (35%) of the patients, and agenesis was found in three (4%) of the patients. Diseases that presented with corpus callosum agenesia were lissencephaly 3 and Lujan–Fryns syndrome. Although these diseases were not primary white matter diseases, they presented with white matter abnormalities or delayed myelination fulfilling the inclusion criteria. Abnormal signal abnormalities of the corpus callosum were noted in seven (8%) of the patients.

### Patterns of abnormal myelination

**FIGURE 4:**
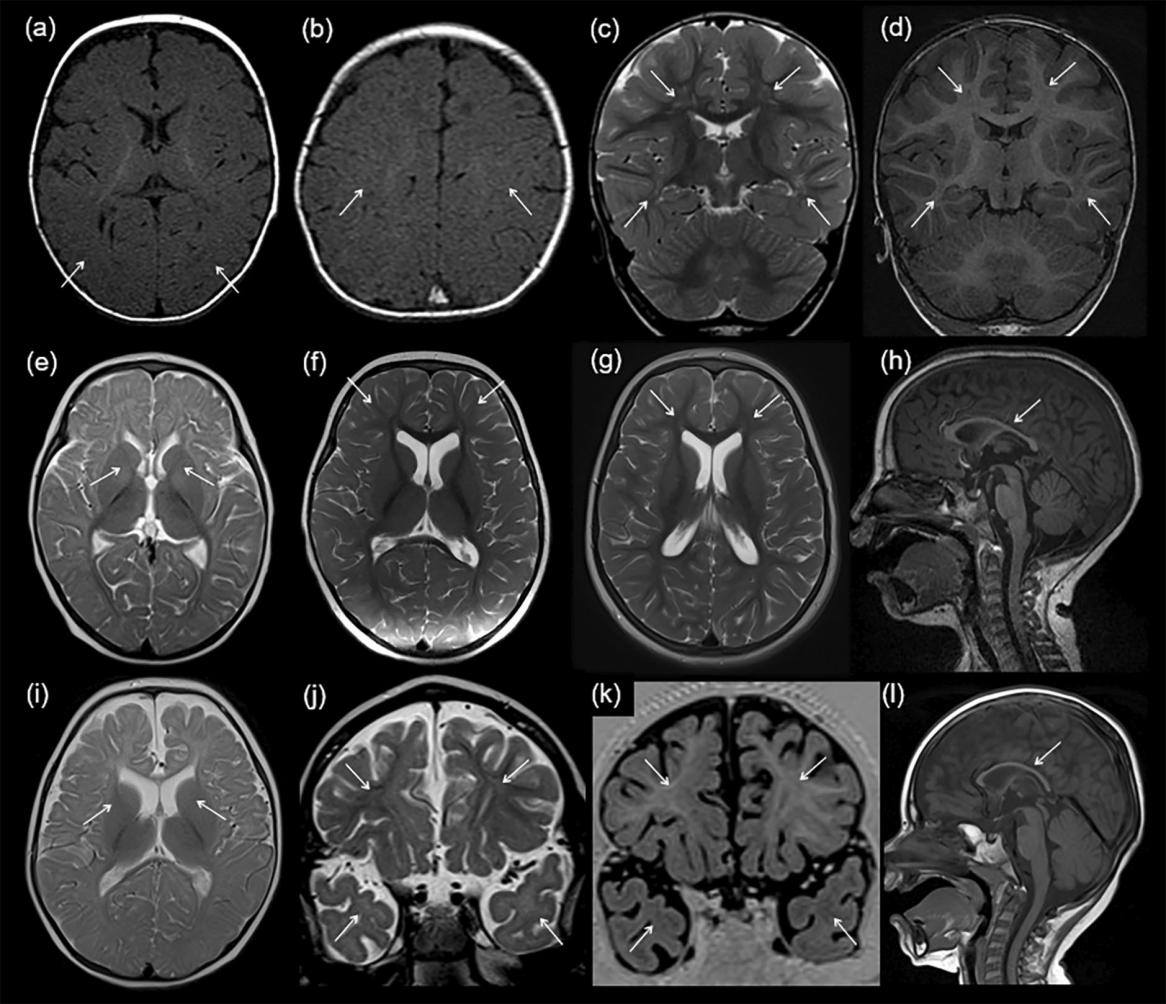
Mild hypomyelination of the diseases (early infantile epileptic encephalopathy 44 [DEE44], neurodegeneration with brain iron accumulation 5 [NBIA5], and X‐linked intellectual disability‐1 [MRX1]) previously not associated with hypomyelination. Axial T1‐weighted images of a 5‐month‐old patient with DEE44 do not present myelination of deep subcortical occipital white matter (a, arrows) or mid‐frontal white matter (b, arrows) as expected at this age. Follow‐up MRI of this patient with DEE44 at the age of 4 years shows slightly patchy frontal and temporal hypomyelination on coronal T2‐weighted (c, arrows) and T1‐weighted (d, arrows) images. Axial T2‐weighted image of a patient with NBIA5 at the age of 10 months does not show myelination of the anterior limbs of the internal capsule (e, arrows). At 22 months of age, maturation has progressed but there is still slight frontal hypomyelination on axial T2‐weighted image (f, arrows). At 3 years 8 months of age this T2‐weighted image of this patient with NBIA5 (g and h) shows patchy signal intensity in the anterior frontal white matter suggesting mild hypomyelination (g, arrows), and the sagittal T1‐weighted image demonstrates thinning of the corpus callosum (h, arrow). A patient with MRX1 at the age of 10 months lacks myelination of the anterior limbs of the internal capsule (i, arrows). At 2 years 9 months of age this patient with MRX1 presents hypomyelination of frontal and temporal white matter on the coronal T2‐weighted image (j, arrows) and T1‐weighted inversion recovery coronal image (k, arrows), and thinning of the corpus callosum on the sagittal T1‐weighted image (l, arrow).

Abnormal myelination was observed in 20 out of 83 patients (24%). All 11 patients with delayed myelination had only one brain MRI scan available. The median age at the diagnosis for delayed myelination was 14 months (IQR = 19 months), and 33 months for permanent hypomyelination. Hypomyelination was found in seven different diseases: Salla disease, 18q deletion syndrome, Cockayne syndrome (type B), mucolipidosis II, neurodegeneration with brain iron accumulation (NBIA5), developmental and epileptic encephalopathy 44 (DEE44), and X‐linked intellectual disability 1 (MRX1) (Figure 4 and Figure S1).

### Radiological findings of the midbrain and pontocerebellar structures

Midbrain, brainstem, pontine, and dentate nuclei abnormalities were separately relatively uncommon but often appeared simultaneously. Symmetrical signal abnormalities in the midbrain were found in eight (10%) of the patients. Pontine T2 signal abnormalities were found in seven (8%) and medulla oblongata involvement in seven (8%) of the patients. Symmetrical T2 signal abnormalities in dentate nuclei were encountered in six (7%) of the patients. Cerebellar, midbrain, and brainstem abnormalities were mostly related to mitochondrial gLEs (Figure 1).

### Stroke‐like MRI findings of mitochondrial GWMDs

Patients with SURF1‐related Leigh syndrome, multiple acyl‐CoA dehydrogenase deficiency (MADD), MELAS, and mitochondrial recessive ataxia syndrome (MIRAS) were the only patients presenting restricted diffusion (Figure S1).

### Abnormal MRI findings of basal ganglia, ventriculomegaly, and atrophy

Signal abnormalities of the basal ganglia were found in putamen (*n* = 3, 4%), globus pallidus (*n* = 5, 6%), and caudate nucleus (*n* = 3, 4%). Signal abnormalities of the thalamus were found in six (7%) of the patients. Grey matter heterotopia (*n* = 2, 2%) and cerebellar cortical dysplasia (*n* = 3, 4%) were rare in this cohort, found only in cases of Walker–Warburg syndrome (MDDGA3) and autosomal dominant intellectual disability 13 (MDR13). Ventriculomegaly due to white matter atrophy was noted in 18 (22%) of the patients. The most common locations of cerebral cortical atrophy were in the frontal (*n* = 12, 14%) and temporal (*n* = 11, 13%) lobes.

### Calcifications

Calcifications were detected in one patient with MELAS and in all patients with CRMCC1 (Figure 2).

### Absence of white matter signal abnormalities

In 11 (13%) of the cases, notable white matter or brain abnormalities were not present. These included all patients with leukoencephalopathy, progressive, with ovarian failure (LKENP, *n* = 2), Fabry disease (*n* = 1), early infantile epileptic encephalopathy 34 (EIEE34, *n* = 1), and carbamoyl phosphate synthetase I deficiency (CPS 1 deficiency, *n* = 1). Normal white matter signal intensity was presented in 1 out of 6 patients with X‐ALD, 1 out of 2 patients with infantile‐onset spinocerebellar ataxia (IOSCA), 1 out of 3 patients with CRMCC1, 1 out of 4 patients with SCBMS, and 2 out of 3 patients with MELAS.

## DISCUSSION

This longitudinal population‐based cohort study presents both typical and atypical brain MRI patterns of known and recently described GWMDs as a diagnostic tool in practice and gives comprehensive insight into the radiological characteristics of GWMDs in a Northern Finnish population (Figures 1, 2, 3, 4). There is a unique prevalence of GWMDs in Finland^10^ because of the enrichment of certain rare variants due to isolation, genetic drift, and population bottlenecks.^18^, ^19^ Similar special prevalences of GWMDs, for example Canavan and Tay–Sachs diseases, with reduced genetic diversity have been reported in the descents of Ashkenazi Jews.^20^ This study also showed that certain brain MRI pattern signs in GWMDs have unique characteristics that are crucial when suspecting and diagnosing these disorders. Furthermore, improved diagnostics with genetic testing and modern MRI techniques increase knowledge of these rare diseases and aid in finding novel GWMDs.

Concerning hypomyelinating disorders in the study cohort, Salla disease and 18q deletion syndrome represented classical hypomyelinating disorders with typical MRI findings. Interestingly, hypomyelinating disorders that are the most common in global settings, such as Pelizaeus–Merzbacher disease and Pelizaeus–Merzbacher‐like disease, were absent in this cohort.^21^ This may be because of enrichment of specific variants in the Finnish population.^18^ In addition to classical hypomyelinating disorders, brain MRI revealed hypomyelination in NBIA5, EIEE44, mucolipidosis II, and MRX1 (Figures 1 and 4).

Classical leukodystrophies were relatively uncommon (17%) in the study cohort, and many demonstrated rare or atypical MRI findings. The only patient with Alexander disease presented a rare and atypical neonatal phenotype in both imaging and clinical settings, which has been reported previously. The predominant imaging findings were progressive ventricular enlargement due to aqueductal stenosis caused by enlargement of tectum, oedema, and signal abnormalities of the deep grey nuclei and tectum, and signal abnormalities of the brainstem and midbrain.^22^

X‐ALD was the most common classical leukodystrophy in this cohort. MRI demonstrated several known patterns of cerebral adrenoleukodystrophy (cALD). Two of the patients presented the common T2 hyperintense lesions in both parietal and occipital white matter, with T2 hyperintensity in the splenium of the corpus callosum. Two patients presented milder white matter signal abnormalities and one patient also had T2 signal abnormalities in the midbrain and pons. MRI of one of the patients was considered normal, representing an X‐ALD patient without cALD. The patients with globoid cell leukodystrophy (Krabbe disease) and metachromatic leukodystrophy (MLD) had classical MRI findings.^6^ The only patient with leukoencephalopathy with brainstem and spinal cord involvement and lactate elevation (LBSL) had confluent supratentorial white matter pathology in addition to classical infratentorial and spinal cord white matter pathology involvement.^6^

The corpus callosum was abnormal in a significant number of diseases and among all subtypes of GWMDs. Terminologies describing a corpus callosum that appears thin but with all its anatomic parts formed vary in the literature. The terms hypoplasia, thinning, atrophy, and hypoplasia with partial agenesis or dysplasia have all been used, most likely with redundancy.^23^ In GWMDs, thinning of the corpus callosum may also be a result of white matter pathology, hypomyelination, or degeneration, and it may not be true congenital hypoplasia.^24^ Because of a wide spectrum of terminology, we combined hypoplasia and thinning of the corpus callosum as a single variable in this study (Figure 3). In hypomyelinating disorders, thinning of the corpus callosum has been a well‐known MRI finding.^24^ However, hypoplasia or thinning of the corpus callosum was also found in 16 diseases other than hypomyelinating GWMDs in this study (Figure 3). A characteristic brain MRI finding of the previously reported FINCA disease in this cohort was a thin corpus callosum (Figure 2).^14^ Other brain abnormalities of the FINCA disease included cortical atrophy and ventriculomegaly.^14^, ^25^

In the patients with SCBMS, the corpus callosum was thin, suggesting hypoplasia, as previously reported.^15^ In addition to corpus callosum hypoplasia, previous studies of SCBMS reported cortical atrophy of the temporal and occipital lobes, occipital white matter signal abnormalities, and volume loss of the white matter.^26^ In this cohort, 3 out of 4 patients with SCBMS demonstrated these imaging findings despite the great variation in the age at which the MRI was done (1 year, 4 years, and 8 years of age). Thus, corpus callosum abnormalities, such as hypoplasia in addition to atrophy and T2 signal abnormalities in the occipital region, are considered typical radiological pattern signs of SCBMS (Figure 2).^15^, ^26^

T2 signal abnormalities in deep grey matter were encountered in all subtypes of GWMDs, and except for FINCA disease, the thalami were always involved.^14^ T2 hyperintensities of the dentate nuclei were found in Krabbe disease, Alexander disease, Leigh syndrome, MADD, and CRMCC1. Cerebellar white matter was relatively rarely involved in the study cohort, since abnormal T2 hyperintensities in cerebellar white matter were interpreted only in Kearns–Sayre syndrome, MADD, and CRMCC1. Progressive cerebellar atrophy with bilateral, periventricular, and multifocal T2 signal abnormalities of the frontal and parietal lobes was found in three patients with ATXPC associated with haematologic disorders due to the pathogenic variants in the *SAMD9L* gene (Figure 2).^12^

In addition to white matter abnormalities, the presence of calcifications and cysts with contrast enhancement may be a crucial pattern sign for the diagnosis of a specific GWMD, as exemplified by CRMCC1, also known as Coats plus syndrome.^13^ This study included three patients with CRMCC1‐related leukoencephalopathy presenting as T2 hyperintensities in the basal ganglia, thalami, parieto‐occipital white matter, and brain stem. Other typical features in neuroimaging are intracranial calcifications and cysts, which often show ring‐like contrast enhancement (Figure 2).^13^, ^27^

### Limitations of the study

To our knowledge, this is the largest Finnish cohort study on GWMDs that includes the MRI data of each patient involved. Nevertheless, the number of patients was relatively small (*n* = 83), with all cases considered rare diseases, making it difficult to establish encompassing imaging patterns of a single disease among this heterogeneous group of GWMDs. One of the inclusion criteria for the study was available brain MRI data. Thus, some patients with known or suspected GWMD were excluded if no brain MRI data were available. Brain MRIs were normal in 11 (13%) of the patients because of their young age at the time of imaging, given treatments, or healing of white matter lesions as a part of the natural course of disease. In many cases, single GWMD was found in only one or two patients, making the generalization of brain MRI findings challenging. Imaging patterns were collected from a relatively small but specific population, posing both limitations and strengths for the study. In our cohort, a combination of comprehensive genetic testing and detailed clinical evaluation was used to confirm the diagnosis in all cases before the secondary more detailed brain MRI analysis performed as a part of this study. Understanding the MRI patterns and thus diagnostics can be improved by learning the principles of the more precise classification of leukodystrophies based on molecular and cellular pathology.^5^ Furthermore, knowledge of imaging patterns will be beneficial in creating diagnostical applications based on artificial intelligence and machine learning.^28^

### Conclusion

This study provides an updated diagnostic tool for interpreting radiologists to analyse brain MRIs of genetic disorders with white matter abnormalities. Since diagnostics, including exome or genome sequencing, continuously add knowledge on novel rare diseases, reporting MRI scans of these diseases becomes beneficial, as radiologists may be the first physicians to suspect or diagnose GWMD.

## CONFLICT OF INTEREST STATEMENT

The authors have no conflicts of interest to declare.

## Supporting information


**Appendix S1:** Research data sheet of brain MRI findings.

**Appendix S2:** Brain MRI findings of genetic disorders with white matter abnormalities in the cohort.

**Figure S1:** Examples of MRI patterns in the cohort.

## Data Availability

The data supporting the findings of this study are available from the corresponding author upon reasonable request. Due to privacy and ethical restrictions, clinical patient data are not available. The authors declare that the submitted work is not being considered for publication anywhere else and has not been previously published.
