# Supplementary material for: Brain MRI findings in paediatric genetic disorders associated with white matter abnormalities
Source: Dev Med Child Neurol. 2024 Jul 30;67(2):186–94. doi: 10.1111/dmcn.16036 (PMC11695792; doi:10.1111/dmcn.16036)
Supplement: Supplementary file 1 — Appendix S1: Research data sheet of brain MRI findings. [file DMCN-67-186-s003.pdf]

**Subject number:**<br>Date of head MRIs:		Finding number	Symmetry	T+	Progression (dt)	Improvement (dt)
Myelination						
	Normal (1)					
	Delayed (2)					
	Absent (3)					
	Permanent hypomyelination (MRIs at least 6 mo apart) (4)					
White matter abnormalities						
	Periventricular (1)					
	Subcortical (2)					
	Diffuse cerebral (3)					
	Focal (4)					
	Multifocal (5)					
	Confluent (6)					
	Cystic cerebral white matter degeneration (7)					
	Frontal (8), parietal (9), occipital (10), temporal (11)					
	Oedema (12)					
	Atrophy (13)					
	T1WI hypointensity (14)					
	T1WI isointensity (15)					
	T1WI hyperintensity (16)					
	T2WI hypointensity (17)					
	T2WI isointensity (18)					
	T2WI hyperintensity (19)					
Basal ganglia and limbic system						
	Abnormality of putamen (1), globus pallidus (2), caudate nucleus (3), thalamus (4), fornix (5), amygdala (6), hippocampus (7), mamillary bodies (8)					
	T2WI hyperintensity (19)					
	Oedema (10)					
	Atrophy (11)					
Corpus callosum						
	Thinning (1)					
	Hypoplastic (2)					
	Agenesis (3)					
	T2WI hyperintensity (4)					
Midbrain and tectum abnormalities						
	T2WI hyperintensity (1)					
	Atrophy (2)					
	Oedema (3)					
Cerebellar abnormalities						
	T2WI hyperintensity: lobes (1), vermis (2)					
	hyperintensity of cerebellar nuclei (dentate nucleus) (3)					
	Lobar hypoplasia (4), hypoplasia of the vermis (5)					
	Progressive atrophy (6)					
	Oedema (7)					
Brainstem and pons abnormalities						
	Brainstem (1), pons (2)					
	Atrophy (3)					
	Hypogenesis (4)					
	T2WI hyperintensity (5)					
	Oedema (6)					
CSF spaces						
	Dilation due to hydrocephalus (1)					
	Dilation due to white matter atrophy (2)					
	Dilation due to cerebellar or brain stem atrophy (3)					
Grey matter abnormalities						
	Cell proliferation disorder (e.g. microcephaly, megalencephaly) (1)					
	Neuronal migration disorder (e.g. heterotopia, cobblestone cortex, lissencephaly, pachygyria) (2)					
	Organization disorder (eg. dysplasia, schizencehaly, polymicrogyria) (3)					
