# Supplementary figures and images for: Brain MRI findings in paediatric genetic disorders associated with white matter abnormalities
Source: Dev Med Child Neurol. 2024 Jul 30;67(2):186–94. doi: 10.1111/dmcn.16036 (PMC11695792; doi:10.1111/dmcn.16036)

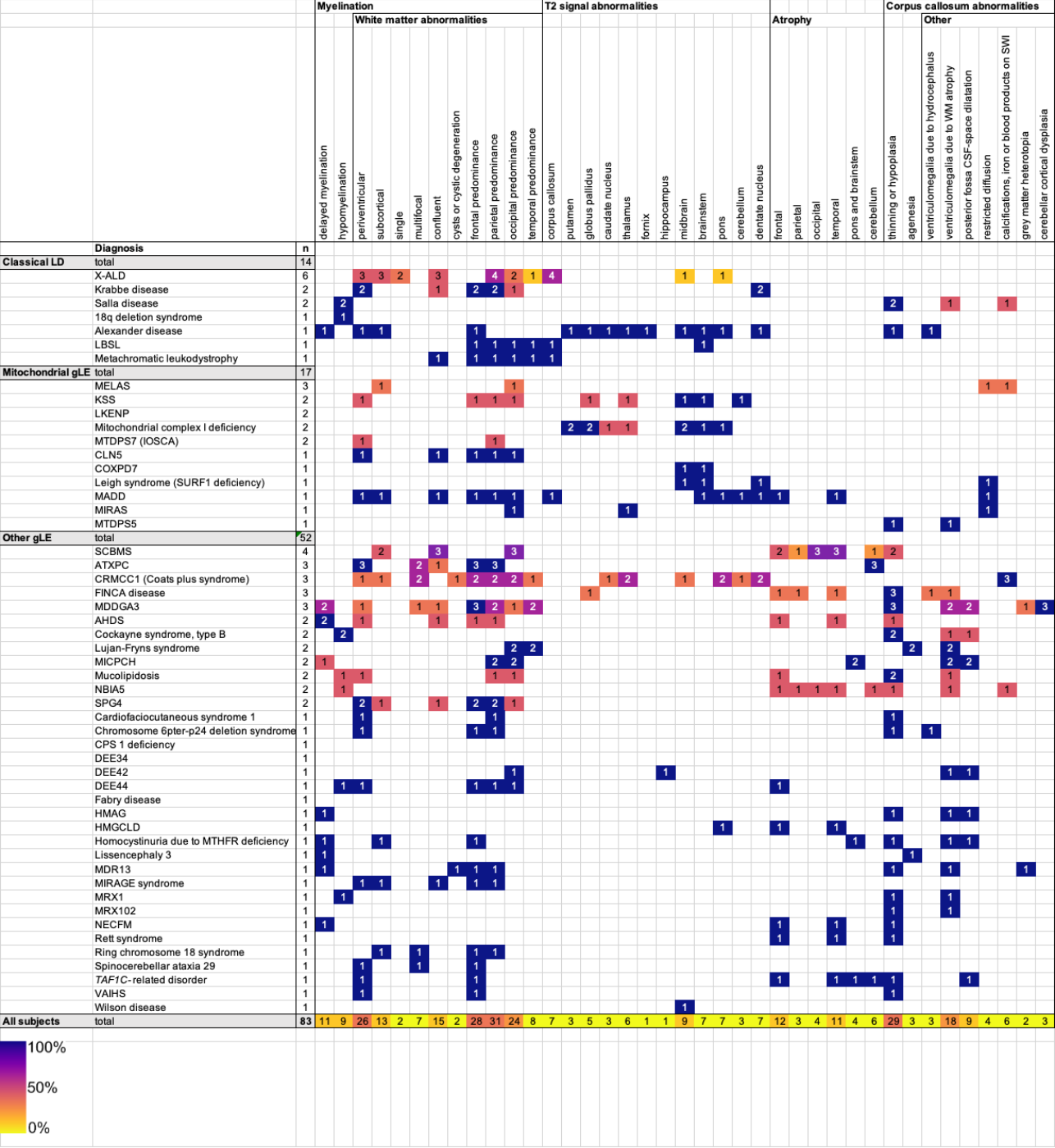

Supplement: Supplementary file 2 — Appendix S2: Brain MRI findings of genetic disorders with white matter abnormalities in the cohort. [file DMCN-67-186-s001.pdf]
