# Supplementary material for: Brain MRI findings in paediatric genetic disorders associated with white matter abnormalities
Source: Dev Med Child Neurol. 2024 Jul 30;67(2):186–94. doi: 10.1111/dmcn.16036 (PMC11695792; doi:10.1111/dmcn.16036)

Figure S1: Examples of MRI patterns in the cohort. Axial T2 fluid-attenuated inversion recovery image of an 8-year-old patient with X-linked adrenoleukodystrophy shows symmetric confluent white matter signal hyperintensity in the parieto-occipital lobes (long arrows in a) and splenium of the corpus callosum (short arrows in a). Axial T2-weighted images show hypomyelination in a 13-year-old patient with Salla disease (b) and 2 years and 9 months old patient with 18q deletion syndrome (c). Axial T2-weighted image (d), diffusion weighted image (e), and apparent diffusion coefficient map (f) of a 9-year-old patient with mitochondrial encephalomyopathy with lactic acidosis and stroke-like episodes show swollen and T2-hyperintense cortex and subcortical white matter of the left occipital lobe parasagittally (arrow in d). There is relatively subtle restricted diffusion, suggesting cytotoxic oedema (arrows in e and f). Axial T2-weighted image (g) demonstrates bilateral dorsomedial thalamic T2-hyperintensities (arrows in g), and diffusion weighted image (h), and apparent diffusion coefficient map (i) show symmetrical restricted diffusion in the occipital lobes (arrows in h and i) of a 15-year-old patient with mitochondrial recessive ataxia syndrome. Axial T2-weighted images (j-l) of a patient with SURF1 deficiency-related Leigh syndrome at the age of 11 months present symmetrical abnormal T2-hyperintensities in the medulla oblongata (arrows in j), midbrain (arrows in k), and thalami (arrows in l).

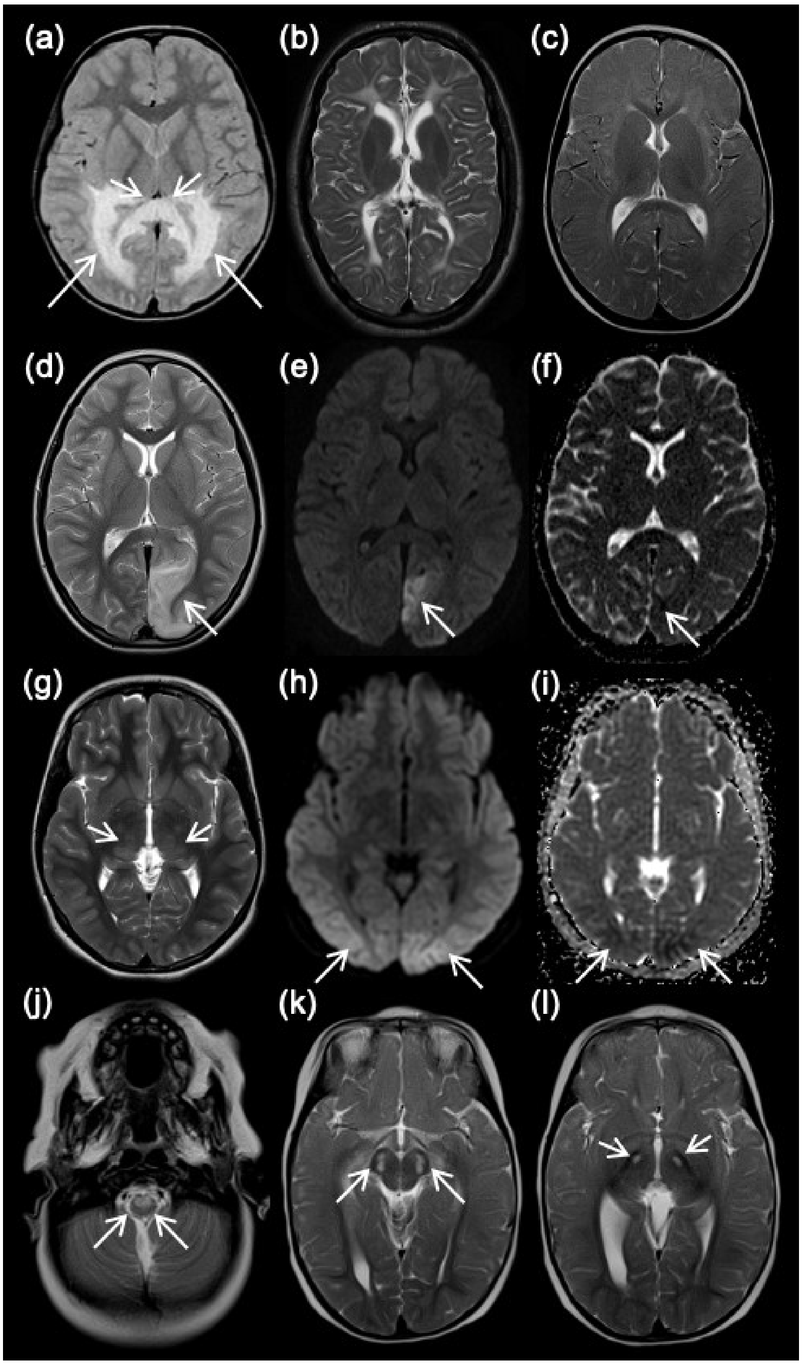

Supplement: Supplementary file 3 — Figure S1: Examples of MRI patterns in the cohort. [file DMCN-67-186-s002.pdf]
